# Detecting the Differences in Responses of Stomatal Conductance to Moisture Stresses between Deciduous Shrubs and *Artemisia* Subshrubs

**DOI:** 10.1371/journal.pone.0084200

**Published:** 2013-12-30

**Authors:** Qiong Gao, Mei Yu, Chan Zhou

**Affiliations:** 1 Landscape Analysis and Modeling Group, National Laboratory of Earth Surface Processes and Resources Ecology, Beijing Normal University, Beijing, China; 2 Institute for Tropical Ecosystem Studies, University of Puerto Rico - Rio Piedras, San Juan, Puerto Rico, United States of America; 3 College of Life Sciences, Liaoning University, Shenyang, China; The Ohio State University, United States of America

## Abstract

Shrubs and subshrubs can tolerate wider ranges of moisture stresses in both soil and air than other plant life forms, and thus represent greater nonlinearity and uncertainty in ecosystem physiology. The objectives of this paper are to model shrub/subshrub stomatal conductance by synthesizing the field leaf gas exchanges data of 24 species in China, in order to detect the differences between deciduous shrubs and *Artemisia* subshrubs in their responses of stomatal conductance to changes in the moisture stresses. We revised a model of stomatal conductance by incorporating the tradeoff between xylem hydraulic efficiency and cavitation loss risk. We then fit the model at the three hierarchical levels: global (pooling all data as a single group), three functional groups (deciduous non-legume shrubs, deciduous legume shrubs, and subshrubs in *Artemisia* genus), and individual observations (species × sites). Bayesian inference with Markov Chain Monte Carlo method was applied to obtain the model parameters at the three levels. We found that the model at the level of functional groups is a significant improvement over that at the global level, indicating the significant differences in the stomatal behavior among the three functional groups. The differences in tolerance and sensitivities to changes in moisture stresses are the most evident between the shrubs and the subshrubs: The two shrub groups can tolerate much higher soil water stress than the subshrubs. The analysis at the observation level is also a significant improvement over that at the functional group level, indicating great variations within each group. Our analysis offered a clue for the equivocal issue of shrub encroachment into grasslands: While the invasion by the shrubs may be irreversible, the dominance of subshrubs, due to their lower resistance and tolerance to moisture stresses, may be put down by appropriate grassland management.

## Introduction

Dynamic global vegetation models (DGVMs) and regional ecosystem models have been making projections of structures and functions in response to changes in climate and anthropogenic activities [1], [2]. However, there existed great disagreements among the models, largely due to the uncertainties in physiological parameters that control the carbon assimilation into and emission out of ecosystems [3]. Ecosystem model parameters are often derived from plant functional traits, which have been recognized as important links to ecosystem functions, structures, and adaptations [4], [5]. Relationships among maximum leaf photosynthesis, maximum stomatal conductance, specific leaf area, leaf life span, leaf size, and leaf nitrogen have been found from the global leaf traits dataset by means of meta-analyses [6], [7], [8], [9]. Shrubland ecosystem physiology in the sub-humid, semiarid, and arid regions involves great uncertainty because of the superior tolerance to moisture stresses, morphological plasticity, and adaptability [10].

Shrubs have been reported to invade grasslands in many places over the world [10], [11], [12], [13], [14], [15]. Many causal factors have been hypothesized to trigger the processes of shrub encroachment. Among these hypotheses, increased drought frequency and shifted rainfall seasonality/intensity have been considered as major drivers [16], [17]. These hypotheses are based on a common assumption that shrubs are more tolerant to drought than grasses. The parameterized stomatal model used in the Patch Arid Land Simulator (PALS) [18], [19] showed that the shrub stomata can tolerate much more severe soil water stress than the grass stomata. When soil moisture is ample, the grasses showed greater stomata conductance than the shrubs.

Shrubs tend to have deep roots, high ratio of leaf to sapwood areas, low vertical shading, and strong morphological plasticity to adapt to variations in climate and soils [20]. The same shrub species can be phreatophyte in sandy soils, but xerophyte in heavy clayey soils to maintain active when soil water potential lower than −5 MPa [21], [22].

Models of stomatal conductance are important tools to quantify leaf production processes of shrubs, since leaf photosynthesis is co-limited by stomatal conductance and biochemical carboxylation [23]. Many stomatal models exist in the literature, with different mechanistic/empirical assumptions and treatments. Ball et al. [24] proposed a simple empirical model of stomatal conductance as a function of net photosynthesis rates and relative humidity on leaf surface. Another model was developed to count the composite effects of net photosynthesis, vapor pressure deficit, and CO_2_ pressure on stomatal conductance [25]. More mechanistic model of stomatal conductance that considered the transient response of stomatal conductance to changes in driving variables was developed by Buckley et al. [26], [27] Incorporating stomatal conductance with hydrological structure and function of tree canopy showed complex interactions between crown structure and hydrological function [28].

A semi-mechanistic model was developed by Gao et al. [29] to calculate stomatal conductance as a function of soil water potential, vapor pressure deficit in air, intercellular CO_2_ concentration, and light irradiance. The model was revised [30]to consider the cavitation loss of xylem conductance and hydrological capacitance, the model considered the cavitation loss of xylem hydraulic conductivity linearly dependent on xylem water potential, so that
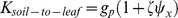
where 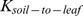 is the apparent soil-to-leaf conductance, *g_p_* is the maximum conductance, and *ζ* is the parameter signifying the linear dependence of 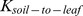 on 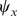, the xylem water potential. However, recent prevailing literatures on functional anatomy of plant xylem of various life forms revealed that the loss of xylem hydraulic conductivity due to cavitation and embolism is significantly related to the vessel diameters [31], [32], [33], [34], [35], [36], [37], [38], despite of some equivocal results with insignificant relationship from a few studies [39], [40]. According to the prevailing literatures, vessels with larger diameter provide greater hydraulic conductivity, however, larger vessels are more prone to cavitation damage by negative pressure inside. Therefore, there exists a tradeoff between the hydraulic conductivity and its sensitivity to cavitation loss. The results of these experiments also indicated that the whole-plant (soil-to-leaf) conductivity decays exponentially with the xylem pressure, rather than the linear assumption in the previous stomatal models.

In this paper, we revised the model of stomatal conductance by Gao et al. [29], [30] according to the prevailing literatures to reflect the tradeoff between the hydraulic efficiency and cavitation resistance. We then fit the revised model using the field diurnal measurements of leaf gas exchange of 24 shrub species in China to test the hypothesis that shrub functional groups differ significantly in their stomatal responses to changes in source (soil) and air moisture stresses. The results indicated that the three shrub functional groups (deciduous non-legume, deciduous legume, and *Artemisia* subshrubs) were significantly different in stomatal behavior. The sensitivities of stomatal conductance of the three shrub functional groups to relevant driving variables were calculated and discussed in the context of experimental evidences.

## Methods

### The Model

We revised the model of stomatal conductance by Gao et al. [30] so that the dependence of soil-to-leaf hydraulic conductance (*K_soil-to-leaf_*) on xylem water potential (*ψ_x_*) is hyperbolic rather than linear, and that the loss of xylem hydraulic conductance depends on the maximum soil-to-leaf conductivity (*g_p_*). Specifically,
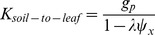
(1)where *λ* is a parameter. Moreover, we assumed *λ* is proportional to *g_p_*, i.e.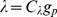, so that
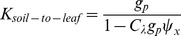
(2)where 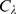 is a constant across all species and all levels of the analysis. This formulation allowed us to balance the hydraulic conductance and the xylem safety, hence the greater the gp, the more vulnerable the xylem system.

In addition to the above changes, the osmotic adjustment is now dependent on net photosynthesis *A_n_* rather than light intensity *I_p_* [41], so that the osmotic pressure of plant leaf and guard cells, *π,* is
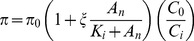
(3)where *π*_0_ is the baseline osmotic pressure (MPa), *C_i_* and *C*_0_ are the intercellular and reference CO_2_ concentrations, respectively, and *ξ* and *K_i_* are parameters. The model now takes similar treatment as Ball et al. [24], [25].

The assumptions in the previous model [30] include: stomatal conductance, *g_s_*, is proportional to the leaf turgor pressure 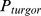,
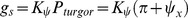
(4)where 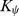 is the apparent compliance of guard cell structure; and transpiration is the product of stomatal conductance and the scaled vapor pressure deficit (*D*), i.e. the absolute vapor pressure deficit (*VPD*) divided by air pressure (*P*).

The mass balance between leaf transpiration and soil-to-leaf flow gives the equation
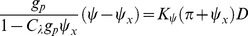
(5)where *ψ* is the soil water potential. Solving the Equation (5) for 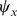 and substituting the result in (4) give the following model
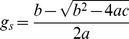
(6)where



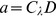
(7)

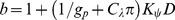
(8)

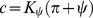
(9)


Equations (3, 6–9) constitute the complete model with 3 basic parameters of 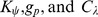, and three osmotic adjustment parameters of 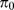, *K_i_*, and ξ.

### Data Collection and Preparation

This analysis used the field diurnal gas exchange data of shrub leaves in northern China (Table 1). Part of the data were collected by the authors and colleagues during 2002–2007, and others were reported in the literature from 1990 to 2003. All the field measurements were done in public lands with free access to researchers. No specific permissions were required since the experiments had no obvious impacts on the sites and did not involve any endangered or protected species. The data from literature were read from tables and charts. Measurement on one species at a site is called an *observation*, and an observation may be done in multiple days. Each observation produced a data table with a number of variables (columns) including stomatal conductance/resistance, net photosynthesis, air pressure, and vapor pressure deficit, at leaf surface. Each record (row) in the data table represented one replicate of measurements on one plant leaf surface at specific hour of the day. There were a total of 43 observations within 80 diurnal measurements involving 24 species in this analysis. Table 1 also listed the approximate geographic location, elevation, instrument used, and data source. Data measured by the authors and colleagues are provided in Table S1.

**Table 1:** List of data sources. PFT – plant functional type, N – number of records.

Observation	Species	PFT	N	Latitude	Longitude	Elevation (m)	Instrument	Reference
1	*Chimonanthus praecox* (L.) Link	DCDS	24					[59]
2	*Mussaenda esquirolli* Levl.	DCDS	47	109.61	26.85	500	LI-6400	Measured
3	*Syringa pekinensis* Rupr.	DCDS	53	115.48	40.02	1100	LI-6400	
4	*Lespedeza bicolor* Turcz.	LEGM	44	115.48	40.02	1100	LI-6400	
5	*Vitex negundo* Linn.	DCDS	53	115.48	40.02	1100	LI-6400	
6	*Caragana pygmaea* (Linn.) DC.	LEGM	176	112.7	42.71	1100	LI-6400	
7	*Caragana korshinskii* Kom.	LEGM	72	112.7	42.71	1100	LI-6400	
8	*Caragana intermedia* Kuang et H. C. Fu	LEGM	72	112.7	42.71	1100	LI-6400	
9	*Artemisia frigida* Willd.	SUBS	122	112.7	42.71	1100	LI-6400	
10	*Artemisia frigida* Willd.	SUBS	82	112.7	42.71	1100	LI-6400	
11	*Artemisia frigida* Willd.	SUBS	124	112.7	42.71	1100	LI-6400	
12	*Caragana microphylla* Lam.	LEGM	144	115.47	42.12	1350	LI-6400	
13	*Artemisia frigida* Willd.	SUBS	169	115.47	42.12	1350	LI-6400	
14	*Salix psammophila* C. Wang et Ch. Y. Yang	DCDS	36	109.19	39.49	1300	LI-6400	
15	*Artemisia ordosica* Krasch.	SUBS	36	109.19	39.49	1300	LI-6400	
16	*Hedysarum fruticosum* Pall.	LEGM	72	109.19	39.49	1300	LI-6400	
17	*Caragana intermedia* Kuang et H. C. Fu	LEGM	72	116.73	43.55	1200	LI-6400	
18	*Salix psammophila* C. Wang et Ch. Y. Yang	DCDS	72	116.73	43.55	1200	LI-6400	
19	*Lespedeza potaninii* Vass.	LEGM	69	116.73	43.55	1200	LI-6400	
20	*Hippophae rhamnoides* Linn.	DCDS	72	116.73	43.55	1200	LI-6400	
21	*Artemisia oxycephala* Kitag.	SUBS	32	116.7	43.63	1200	CI-301 PS	[60]
22	*Paeonia suffruticosa* Andr.	DCDS	36	115.42	39.97	100	CI-301PS	[61]
23	*Caragana intermedia* Kuang et H. C. Fu	LEGM	23	109.85	39.03	1300	LI-6000	[62]
24	*Artemisia ordosica* Krasch.	SUBS	24	109.85	39.03	1300	LI-6000	
25	*Lonicera maackii* (Rupr.) Maxim.	DCDS	13	116.37	39.93	50	CI-301 PS	[63]
26	*Lagerstroemia indica* Linn.	DCDS	13	116.37	39.93	50	CI-301 PS	
27	*Viburnum rhytidophyllum* Hemsl.	DCDS	13	116.37	39.93	50	CI-301 PS	
28	*Sambucus williamsii* Hance	DCDS	13	116.37	39.93	50	CI-301 PS	
29	*Artemisia frigida* Willd.	SUBS	7	120.75	42.88	500	CI-301 PS	[64]
30	*Caragana microphylla* Lam.	LEGM	7	120.75	42.88	500	CI-301 PS	
31	*Hippophae rhamnoides* Linn.	DCDS	48	109.25	36.71	1350	LI-6400	Measured
32	*Caragana korshinskii* Kom.	LEGM	36	109.25	36.71	1350	LI-6400	
33	*Lespedeza daurica* (Laxm.) Schindl.	LEGM	22	109.25	36.71	1350	LI-6400	
34	*Caragana korshinskii* Kom.	LEGM	18	104.85	37.45	1300	LI-6200	[65]
35	*Artemisia ordosica* Krasch.	SUBS	18	104.85	37.45	1300	LI-6200	
36	*Hedysarum scoparium* Fisch. et Mey.	LEGM	13	104.85	37.45	1300	Li-6200	[66]
37	*Caragana korshinskii* Kom.	LEGM	13	104.85	37.45	1300	Li-6200	[67]
38	*Artemisia ordosica* Krasch.	SUBS	13	104.85	37.45	1300	Li-6200	
39	*Caragana korshinskii* Kom.	LEGM	21	104.95	37.33	1300	CI-301PS	[68]
40	*Caragana intermedia* Kuang et H. C. Fu	LEGM	21	109.19	39.49	1300	CI-301PS	
41	*Caragana microphylla* Lam.	LEGM	21	120.92	42.38	1300	CI-301PS	
42	*Alhagi sparsifolia* Shap.	DCDS	42	86.2	38.37	1400	LI-6400	[69]
43	*Calligonum caput-medusae* Schrenk	DCDS	42	86.2	38.37	1400	LI-6400	

To facilitate the analysis, all datasets of the diurnal measurements were converted to the formats and units used by the portable photosynthesis system Licor-6400 (Licor, Nebraska, USA). To avoid the problem of repeated measurements on the same plant leaf, the records of all data tables were averaged for each leaf at a particular hour.

### Procedures of Analysis

The observations were grouped into three functional groups: non-legume deciduous shrubs (DCDS), deciduous legume shrubs (LEGM, mostly in *Caragana* genus), and subshrubs (SUBS, *Artemisia* genus). The LEGM group, with their potential nitrogen fixation capability, may be advantageous over the DCDS when nitrogen is limiting. The subshrubs have characteristics of both woody and herbaceous plants (woody based but herbaceous primary growth).

The above model of stomatal conductance was fitted to the data at three hierarchical levels: observations (OBS, species cross sites), functional groups (PFT), and global (GLB, pooling all data into a single group). Parameter 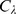 is treated as a super parameter for all the species and observations, so that the tradeoff between conductivity and xylem safety is realized. Model parameters were estimated by means of Bayesian Inference using Gibbs sampling with Markov Chain Monte Carlo (MCMC), implemented in the WinBUGS program [42], [43] run within the free software of R [44].

Fitting the stomatal conductance model required the data for soil water potential which is not available. To get around the problem, we made an additional assumption that soil water potential is approximately constant for each day and the daily soil water potentials were estimated at the observation level. The assumption behind this treatment is that minimizing the difference between the model and the data should also allow us to estimate both model parameters and the unknown daily soil water potential [29], [30]. The estimated daily soil water potentials from the observation level were used at the levels of GLB and PFT.

MCMC requires specification of prior probability distributions for the parameters. On the other hand, it is generally difficult for the iterations to converge to the physiologically meaningful results because of the nature of nonlinear models. We handled this difficulty by providing priors of uniform distributions within the specified physiologically meaningful ranges. This allowed us to place the following bounds to the parameters: 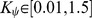 mol H_2_O m^−2^ s^−1^ MPa^−1^, 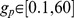
mol H_2_O m^−2^ s^−1^ MPa^−1^, 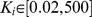 µmol CO_2_ m^−2^ s^−1^,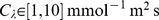, 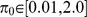 MPa, and 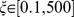. The parameter 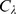 was only fitted at the observation level, so that the parameter at the other two levels (PFT and GLB) was fixed at the mean value from the observational fit. Since the previous studies have recognized the higher drought tolerance of shrubs than other functional types, we placed the bounds of daily soil water potential as 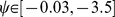 MPa. These bounds were realized in the specification of uniform prior distribution. For example, a statement in the WINBUGS program, ∼dunif (0.01, 1.5) specifies that the prior for the parameter 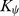 is a uniform distribution between 0.01 and 1.5 mol m^−2^ s^−1^ MPa^−1^. For each level of the hierarchical analysis, the MCMC was iterated for 5,000 times so that almost all parameters can converge.

## Results

### The Statistics of the Fitted Models and Parameters

**Figure 1:**
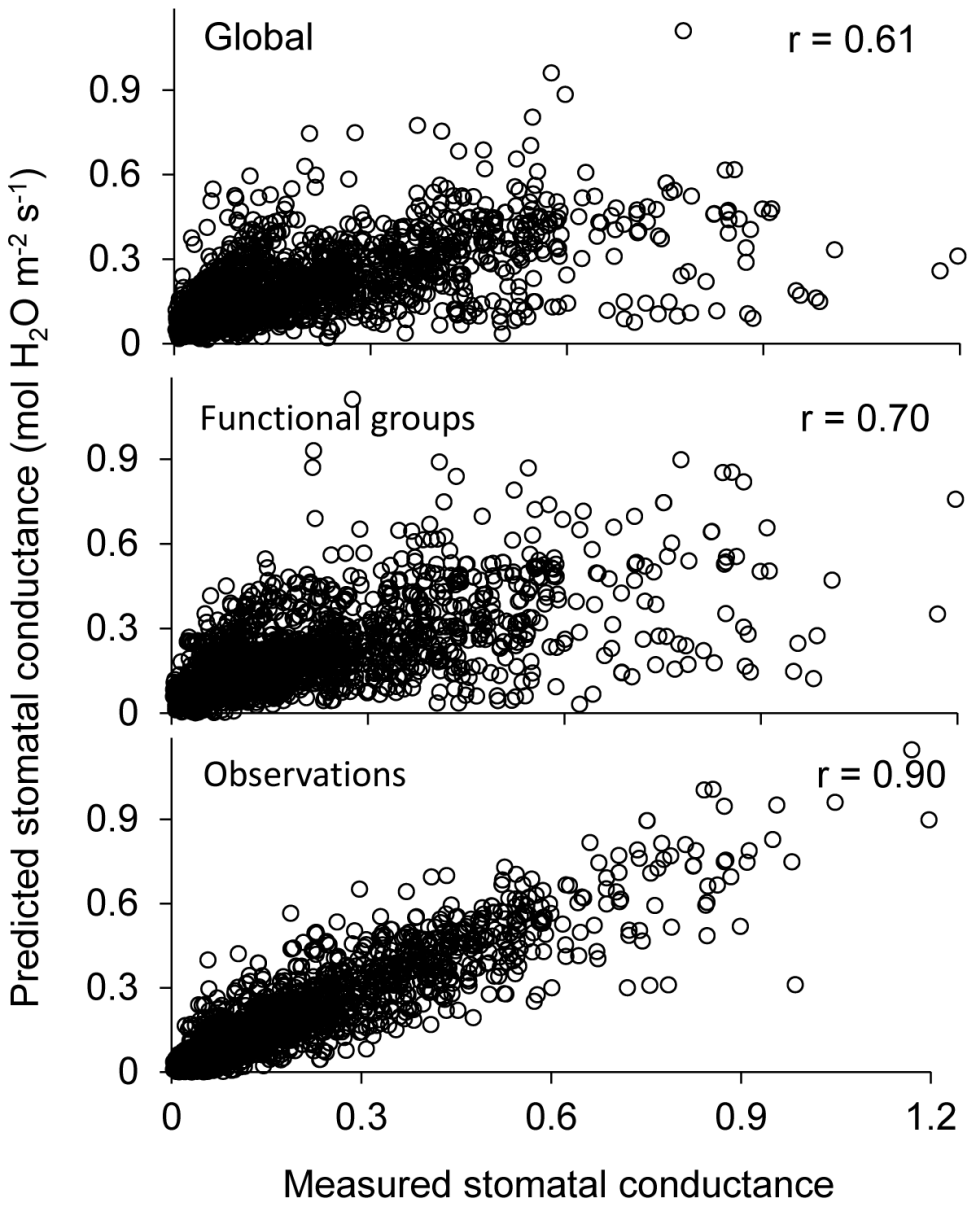
Model predicted vs. measured stomatal conductance at the three hierarchical levels.

Summary statistics (Table 2) for the analysis at the three levels indicate that from global to shrub functional groups to individual observations, the deviances of the models decrease, while the number of parameters increases. The deviance for stomatal model is negative because most measured and predicted stomatal conductance are smaller than 1. Specifically, the deviance decreased from −1,423 to −1,804 to −3,981, and the standard error of the residual decreased from 0.170 to 0.158 to 0.095 mol m^−2^ s^−1^, for GLB, PFT, and OBS levels, respectively. Smaller deviances indicate better fits of the models to the data, in the price of increased model complexity with more parameters. The difference in deviances between any two hierarchical levels has been shown to follow the 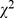 distribution with the degrees of freedom equal to the difference in number of parameters between the two levels. Significant differences in model quality between the two hierarchical levels should be indicated in a significantly large 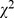 value. The deviance tests (Table 2) among the three levels, viz. PFT vs. GLB, and OBS vs. PFT, indicate that the model for shrub functional groups (PFT) is a significant improvement over the global model (GLB), and that there is significant difference in behavior of stomatal conductance among the three shrub functional groups. Similarly, the model at the observation level is significantly better than the models at the level of functional groups. The trend of improved model fit from global to functional groups to observations is further demonstrated in the increased correlations between the model predictions and measurements (Table 2). Specifically the Pearson’s correlation coefficient between the measured and predicted stomatal conductance increased from 0.61 to 0.70 to 0.90 (Fig. 1) as the analyses went from global to functional groups to observation levels. The plot of the model predicted against the measured stomatal conductance (Fig. 1) showed the progressive improvement of the fitting from coarse to fine granularity.

**Table 2:** Deviances calculated by the WINBUGS, and the Chi-square tests of deviances among the three levels (Global, Functional group, and Observation).

Analysis Level	Global	Functional group		Observation
Deviance	−1,423	−1,804		−3,981
Deviance Information Criterion	−1,485	−1,789		−24,249
Number of Parameters	5	15		296
Standard deviation of error	0.170	0.158		0.095
Difference in deviance	378			2,897
Difference in number of parameters	10			281
p-value of χ^2^ test	<0.0001			<0.0001
Correlation between measured and predicted stomatal conductance	0.61	0.70		0.90

The obtained parameters of the stomatal model at the observation level (Table 3) showed wide ranges of variations across species and sites. Differences in plant and leaf ages, water and nutrient status, instruments used, completeness and accuracy of the data, timing of measurements, and instability of nonlinear algorithms, might all have contributed to these large variations. The super parameter 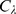 is 3.13±1.66 (m^2^ s mol^−1^).

**Table 3:** Obtained parameters of the stomatal model at individual observation level.

Observation	PFT	*K_ψ_*	*g_p_*	*K_i_*	*π*_0_	*ξ*
1	DCDS	0.088	33.11	295.4	0.95	149.6
2	DCDS	0.552	28.10	326.8	1.30	9.8
3	DCDS	0.380	20.08	324.1	1.02	99.0
4	LEGM	0.454	18.14	330.1	1.02	79.8
5	DCDS	0.297	26.79	329.7	1.02	69.1
6	LEGM	0.591	10.95	330.1	1.54	2.9
7	LEGM	0.639	17.16	333.9	1.04	12.0
8	LEGM	0.734	10.57	329.9	1.20	11.9
9	SUBS	0.228	25.90	345.8	1.41	26.0
10	SUBS	0.508	22.16	338.7	1.09	27.4
11	SUBS	1.084	11.64	345.1	1.51	2.9
12	LEGM	0.927	1.94	362.5	1.39	88.7
13	SUBS	1.461	5.06	369.7	1.95	15.7
14	DCDS	1.034	2.77	302.1	1.39	45.8
15	SUBS	0.986	1.47	227.2	1.16	135.1
16	LEGM	0.576	9.83	41.4	0.94	5.6
17	LEGM	1.034	1.66	67.7	1.26	38.3
18	DCDS	0.460	19.17	308.3	1.12	36.5
19	LEGM	1.307	3.90	219.8	1.74	34.2
20	DCDS	0.526	6.42	45.3	1.04	49.3
21	SUBS	0.282	30.17	316.3	1.54	8.8
22	DCDS	0.263	20.60	304.4	1.06	191.7
23	LEGM	1.407	2.82	26.5	1.35	3.6
24	SUBS	1.482	8.28	331.1	1.98	1.7
25	DCDS	0.322	20.99	305.4	0.71	149.0
26	DCDS	0.345	19.93	311.0	0.76	151.9
27	DCDS	0.311	22.57	308.1	0.77	148.7
28	DCDS	0.180	27.62	313.2	0.85	140.8
29	SUBS	0.311	22.94	314.0	0.82	152.0
30	LEGM	0.262	25.18	305.6	0.78	162.8
31	DCDS	1.090	0.87	27.4	1.43	10.6
32	LEGM	0.845	1.71	319.5	1.55	113.5
33	LEGM	0.246	23.21	271.1	1.21	130.4
34	LEGM	0.565	26.70	325.6	1.31	33.9
35	SUBS	0.599	8.05	303.0	1.17	158.1
36	LEGM	0.551	38.58	320.1	1.38	30.9
37	LEGM	0.452	50.90	341.6	1.69	51.5
38	SUBS	0.807	55.83	314.9	1.86	7.4
39	LEGM	0.236	18.59	298.9	1.15	205.1
40	LEGM	0.636	5.95	323.0	1.43	87.9
41	LEGM	0.157	23.07	317.9	1.27	150.3
42	DCDS	0.745	2.56	325.9	1.50	119.3
43	DCDS	0.407	12.50	309.5	1.32	150.7
Mean		0.613	19.06	283.9	17.36	76.7
Standard deviation		0.373	13.96	93.0	12.95	63.5
Maximum		1.482	62.13	369.7	55.83	205.1
Minimum		0.088	1.04	26.5	0.87	1.7

*C_λ_* is 3.13±1.66 m^2^ s mol^−1^. Units of the parameters: 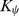, mol m^−2^ s^−1^ MPa^−1^, 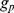, mmol m^−2^ s^−1^ MPa^−1^, *K_i_*, µmol CO_2_ m^−2^ s^−1^, 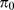, MPa, and *ξ* is dimensionless.

Despite that we used the independent uniform prior distributions for the model parameters, we detected significant negative correlations between the fitted parameters 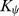 and 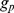 (r = −0.44, p = 0.002), and between 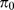 and *μ* (r = −0.44, p = 0.0015), and positive correlations between parameters 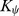 and 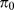 (r = 0.33, p = 0.021), between 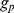 and 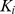 (r = 0.44, p = 0.073). The negative correlation between 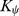 and 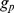 indicated that the stiffness of guard cell structure and efficiency of hydraulic conductance exhibit some kind of coordination. The more efficient vertical xylem transport, the stiffer the guard cell structure to keep the stomata open. It also reflects the balance between stomatal sensitivity to turgor pressure and xylem sensitivity to cavitation.

**Figure 2:**
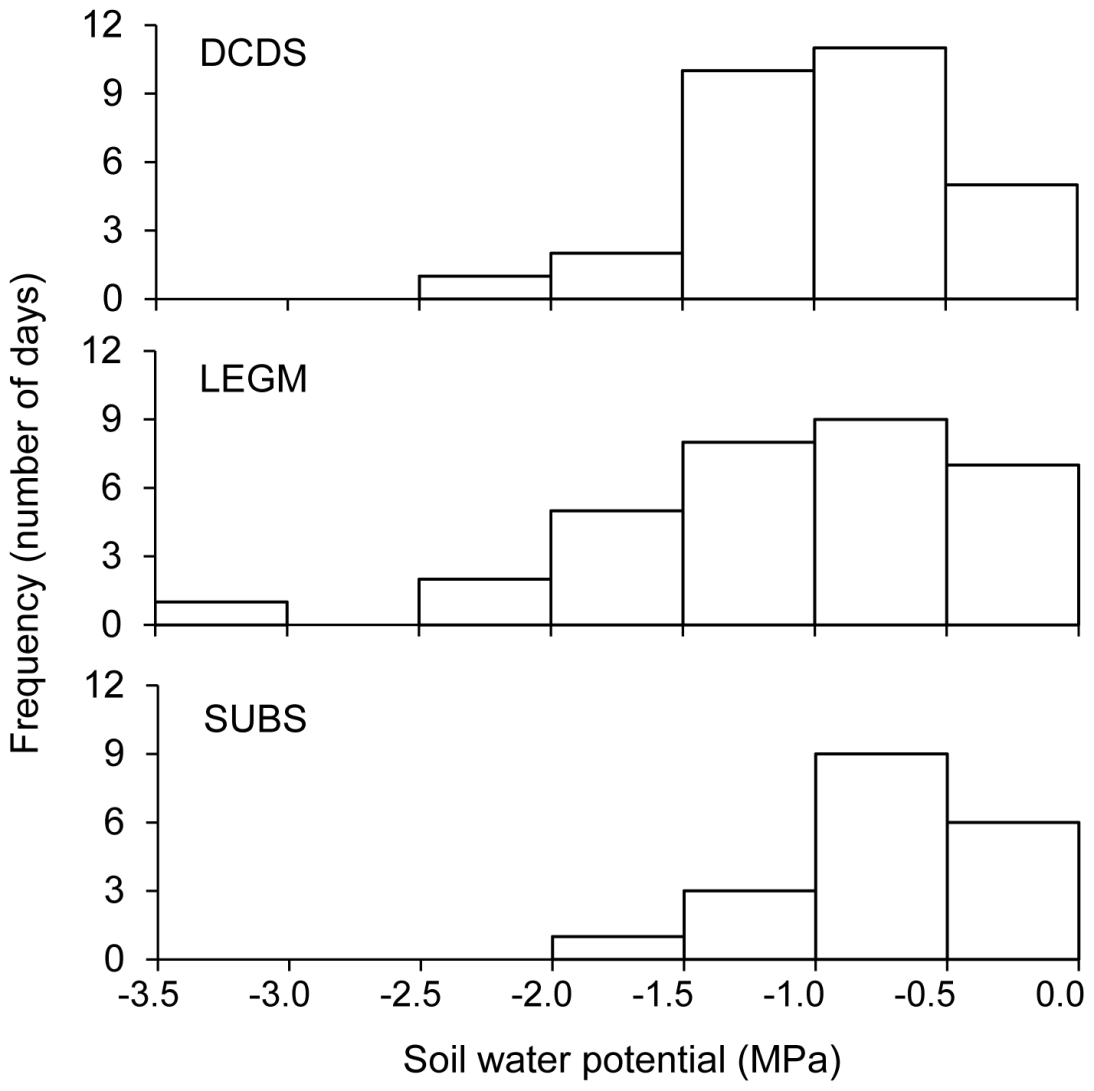
Histograms of the fitted soil water potentials for the three functional groups.

The obtained soil water potentials at the observation level (Fig. 2) were plotted as histograms of the three functional types. We found the mean and standard deviations of the soil water potentials were −0.94±0.49, −1.08±0.66, and −0.73±0.41 MPa for DCDS, LEGM, and SUBS, respectively. The t test to compare the means between LEGM and SUBS resulted in a p-value of 0.024, and the comparison between DCDS and SUBS resulted in a p-value of 0.13. The result indicates that the fitted soil water potentials for the subshrubs are significantly higher than the LEGM shrubs. The means of the soil water potential are not significantly different from each other between the LEGM and DCDS groups. The correlations among the model parameters reflect the complex interactions among various components involved in water transportation from soil to plant leaves [28].

At the functional group level (Table 4), the fitted mean compliance 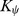 varied from 0.16 to 0.70 to 1.45, reflecting the decreased stiffness of guard cell structure from DCDS to LEGM to SUBS, respectively. However, the maximum potential soil-to-leaf conductance is the greatest for SUBS (5.66 mmol m^−2^ s^−1^ MPa^−1^), but the smallest for LEGM (2.05 mmol m^−2^ s^−1^ MPa^−1^), with DCDS in between (2.57 mmol m^−2^ s^−1^ MPa^−1^).

**Table 4:** Parameters of the stomatal model estimated by the WinBUGS at the global (GLB) and the functional group levels.

Parameter		GLB	DCDS	LEGM	SUBS
*K_ψ_*	mean	0.38	0.16	0.70	1.42
	STD	0.10	0.03	0.27	0.15
***g_p_***	mean	3.60	2.57	2.05	5.66
	STD	3.70	1.19	0.90	2.37
*K_i_*	mean	202.6	198.8	284.0	319.8
	STD	129.5	142.5	130.0	122.8
*π*_0_	mean	1.76	1.91	1.94	1.87
	STD	0.39	0.08	0.17	0.23
*ξ*	mean	25.5	23.8	34.1	9.9
	STD	48.6	15.9	17.3	11.5
**N_day_**	80		24	36	20
**N**	2119		576	916	627
**N_obs_**	43		15	18	10

N is the number of data points involved in the WinBUGS calculation. N_obs_ is the number of observations, and N_day_ the number of diurnal days of measurements.

While 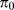 is the baseline osmotic pressure, the combination of 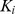 and ξ determines the extent and sensitivity of osmotic adjustment. The three functional groups have similar 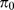 in the range of 1.87 to 1.94 MPa. The DCDS group is shown to have the smallest mean 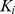 value (198.8 µmol CO_2_ m^−2^ s^−1^), whereas the SUBS has 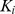 of 319.3. The SUBS has the lowest *ξ* (9.9) followed by 23.8 for DCDS and 34.1 for LEGM. Referring to Equation (3), the efficiency of osmotic adjustment is largely determined by the ratio of *ξ* to 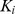 since 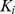 is much greater than the maximum net assimilation (<20 µmol m^−2^ s^−1^) in this case. The obtained model parameters at the global level (Table 4) mostly fall in the range of those at the level of functional groups, except for *π*_0_ of 1.76, which is smaller than any of the three functional groups.

### Behavior of the Stomatal Model

**Figure 3:**
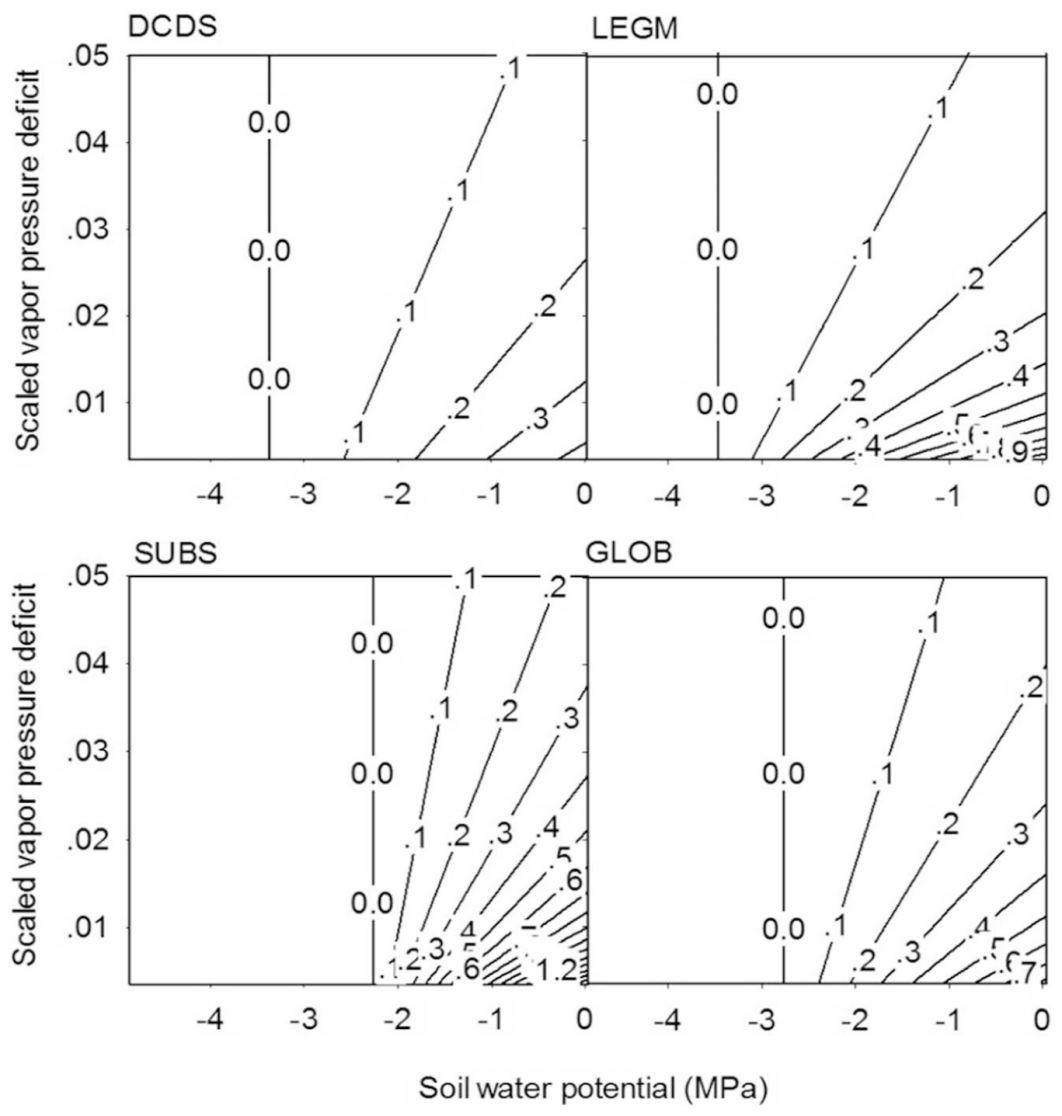
Stomatal conductance (contour lines, mol H_2_O m^−2^ s^−1^) as functions of soil water potential and scaled vapor pressure deficit for the three shrub functional groups, calculated based on the parameters obtained at the level of functional groups: the deciduous non-legume shrubs (DCDS), the deciduous legume shrubs (LEGM), and the *Artemisia* subshrubs (SUBS). The stomatal conductance calculated from the global level model (GLOB) is also plotted as the comparison. Net assimilation *A_n_* is fixed at 6.5 µmol CO_2_ m^−2^ s^−1^.

Based on the model parameters at the levels of PFT and GLB, calculated stomatal conductance was plotted as functions of soil water potential and dimensionless vapor pressure deficit (Fig. 3), with leaf net assimilation fixed at 6.5 µmol m^−2^ s^−1^ (approximately the mean value of net photosynthesis for these groups). Sharp comparison among the three shrub functional groups exists. The lower right corners of the panels depict the maximum stomatal conductance at favorable moisture conditions with *ψ* = −0.033 MPa (approximately field capacity) and *D = *0.0035. At temperature of 30°C at the sea level, *D* of 0.0035 means 91% of relative humidity. At these conditions, the subshrub has the highest stomatal conductance of 1.7 mol m^−2^ s^−1^ followed by the 1.1 of the LEGM group. However, the DCDS group has the much smaller stomatal conductance of 0.44 mol m^−2^ s^−1^. The GLB has the maximum stomatal conductance of 0.83 mol m^−2^ s^−1^, slightly smaller than the average of the three functional groups. The ranking of these values is largely determined by the 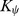 parameter (Table 4). When moisture conditions are favorable, greater guard cell compliance implies greater aperture of stomata, hence greater stomatal conductance.

The subshrub group has lower tolerance to soil water stress than the two deciduous shrub groups, as the stomatal conductance of SUBS closes at the soil water potential of −2.2 MPa. In contrast, the stomatal conductance of DCDS and LEGM groups decrease to zero at −3.2 and −3.5 MPa, respectively. However, the subshrub group has higher tolerance to vapor pressure deficit than the two deciduous shrub groups (Fig. 3). When the soil water potential is high (−0.033 MPa) but the scaled vapor pressure deficit is great (0.05), the subshrub group maintains greater stomatal conductance (0.23 mol m^−2^ s^−1^) than the two deciduous shrub groups (approximately 0.14 mol m^−2^ s^−1^).

The tolerance to soil water stress is primarily determined by the osmotic pressure and the compliance of guard cell structure. The SUBS has low tolerance to soil water stress largely because of the greatest 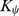 which makes the stomata sensitive to changes in soil water stress.

The spacing and slope of the contour lines (Fig. 3) represent the relative sensitivities of stomatal conductance with respect to soil water stress and vapor pressure deficit. The response of stomatal conductance to vapor pressure deficit is determined by the ratio of 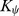 to 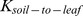, so that greater 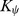 but smaller 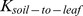 makes the stomatal conductance more sensitive to VPD. A smaller 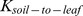 tends to cause insufficient water supply from roots to leaves under greater VPD, and the insufficient water supply decreases the leaf xylem water potential, turgor pressure, and stomatal conductance. A greater 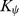 will make stomata more sensitive to the decrease in the turgor pressure, so that the stomatal conductance decreases faster with VPD. The LEGM and SUBS groups have high sensitivity to vapor pressure deficit, largely because the former has the smallest 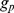 (maximum 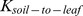), and the latter has the greatest 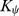 values. The DCDS group has a small 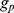 (2.57 mmol m^−2^ s^−1^ MPa^−1^), however, it also has a small 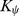 (0.16 mol m^−2^ s^−1^ MPa^−1^), so that the stomatal conductance is not as sensitive as LEGM or SUBS.

**Figure 4:**
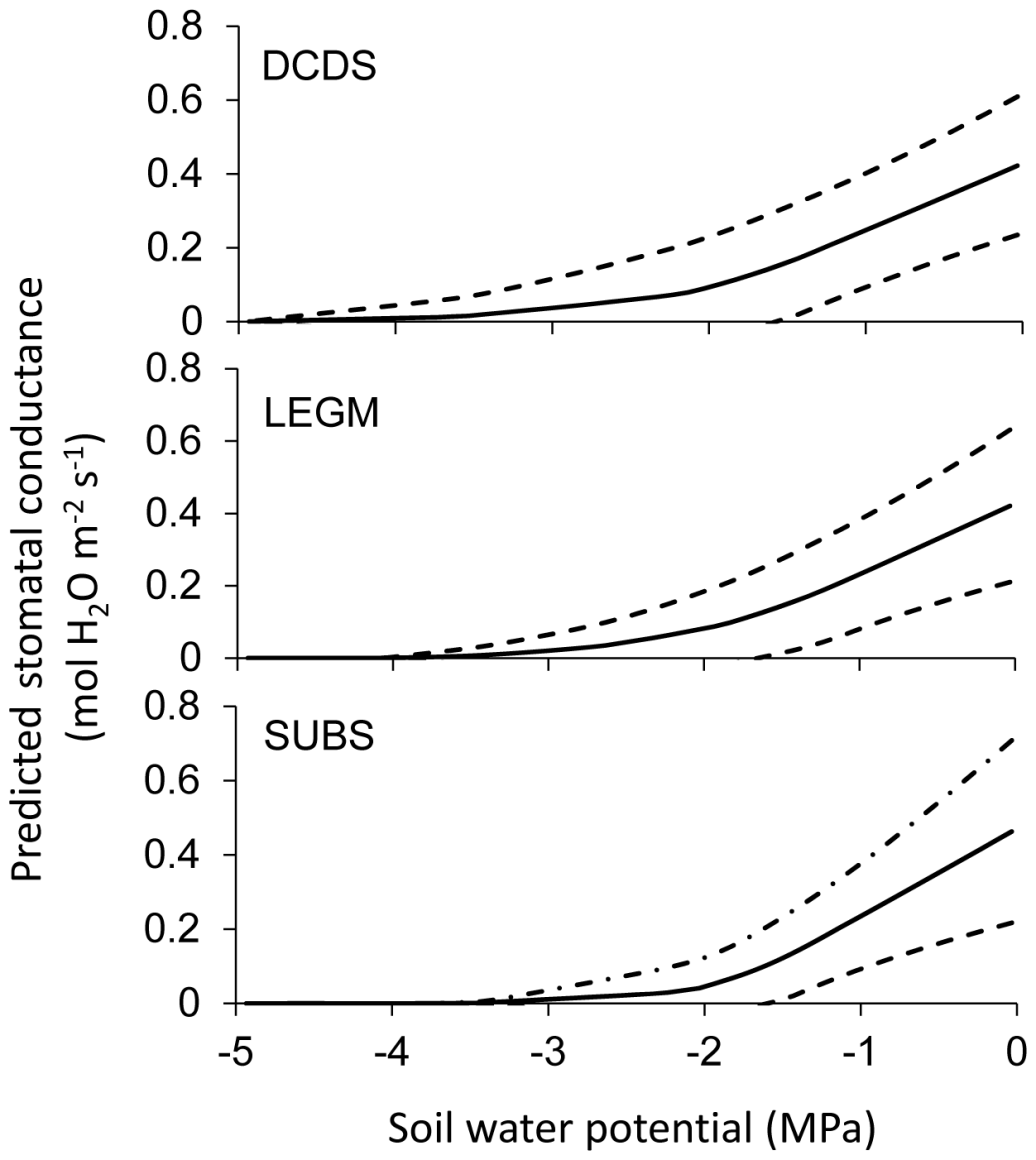
Predicted stomatal conductance as functions of soil water potential based on the parameters at the observation level. The scaled vapor pressure deficit is fixed at 0.03 and the net assimilation at 6.5 µmol CO_2_ m^−2^ s^−1^, for all individual observations. The solid line stands for the mean, and the broken lines indicate one standard deviation below and above the mean.

**Figure 5:**
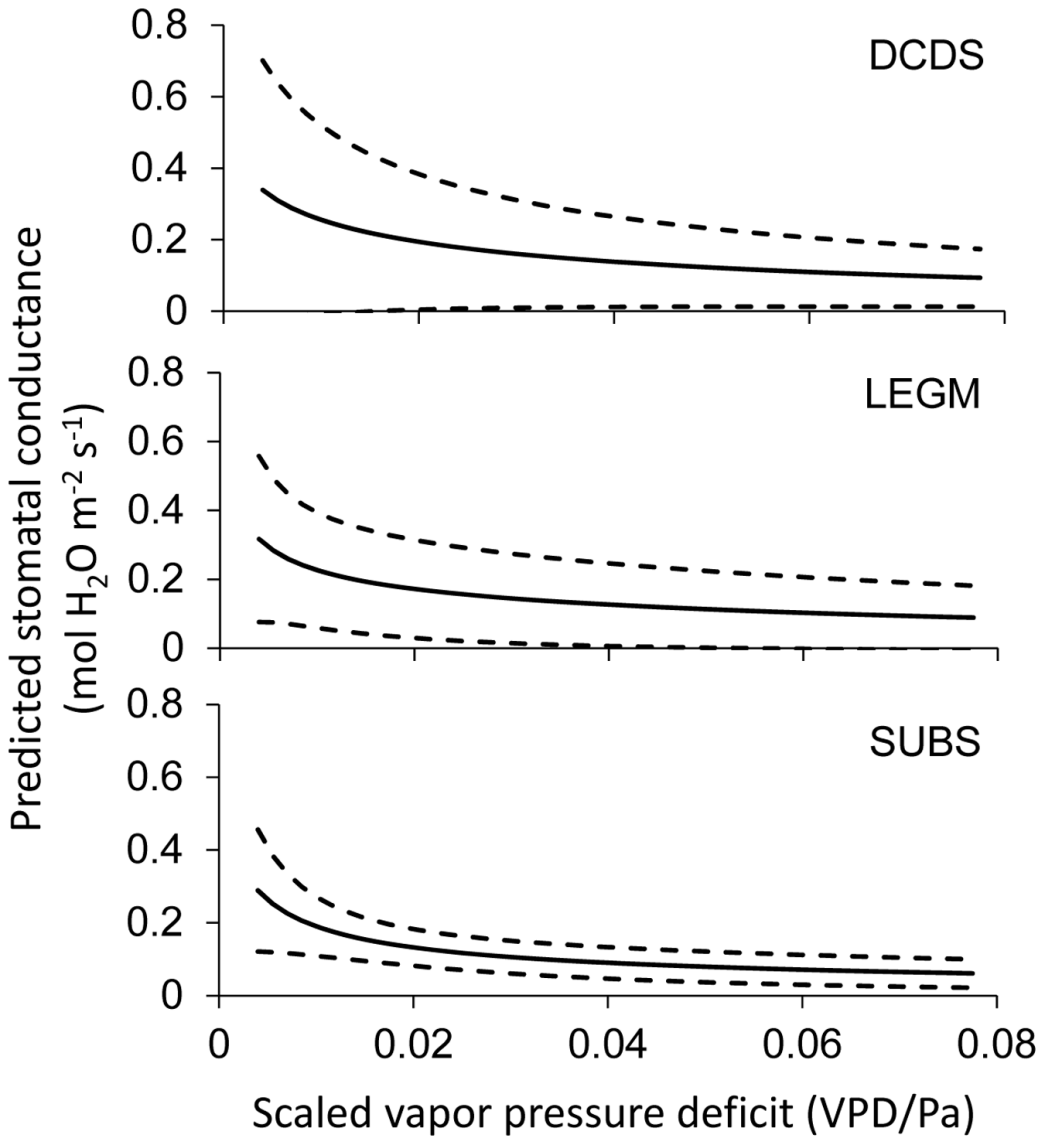
Predicted stomatal conductance as functions of scaled vapor pressure deficit based on the parameters at the observation level. The soil water potential is fixed at −1.0 MPa for all individual observations, and net assimilation is treated as the same as in Fig. 3. The solid line stands for the mean, and the broken lines indicate one standard deviation below and above the mean.

We also used the fitted parameters at the observation level to calculate stomatal conductance of all the observations, and plotted the arithmetic means and one standard deviation below and above against soil water potential (Fig. 4) and scaled vapor pressure deficit (Fig. 5). The results confirmed the findings at the level of functional groups. The average stomatal conductance of DCDS, LEGM, and SUBS close approximately at −4, −3.6, and −3 MPa, respectively, so that the tolerance and sensitivity to soil water stress and vapor pressure deficit of the three functional groups follow the same ranks in Fig. 3.

**Figure 6:**
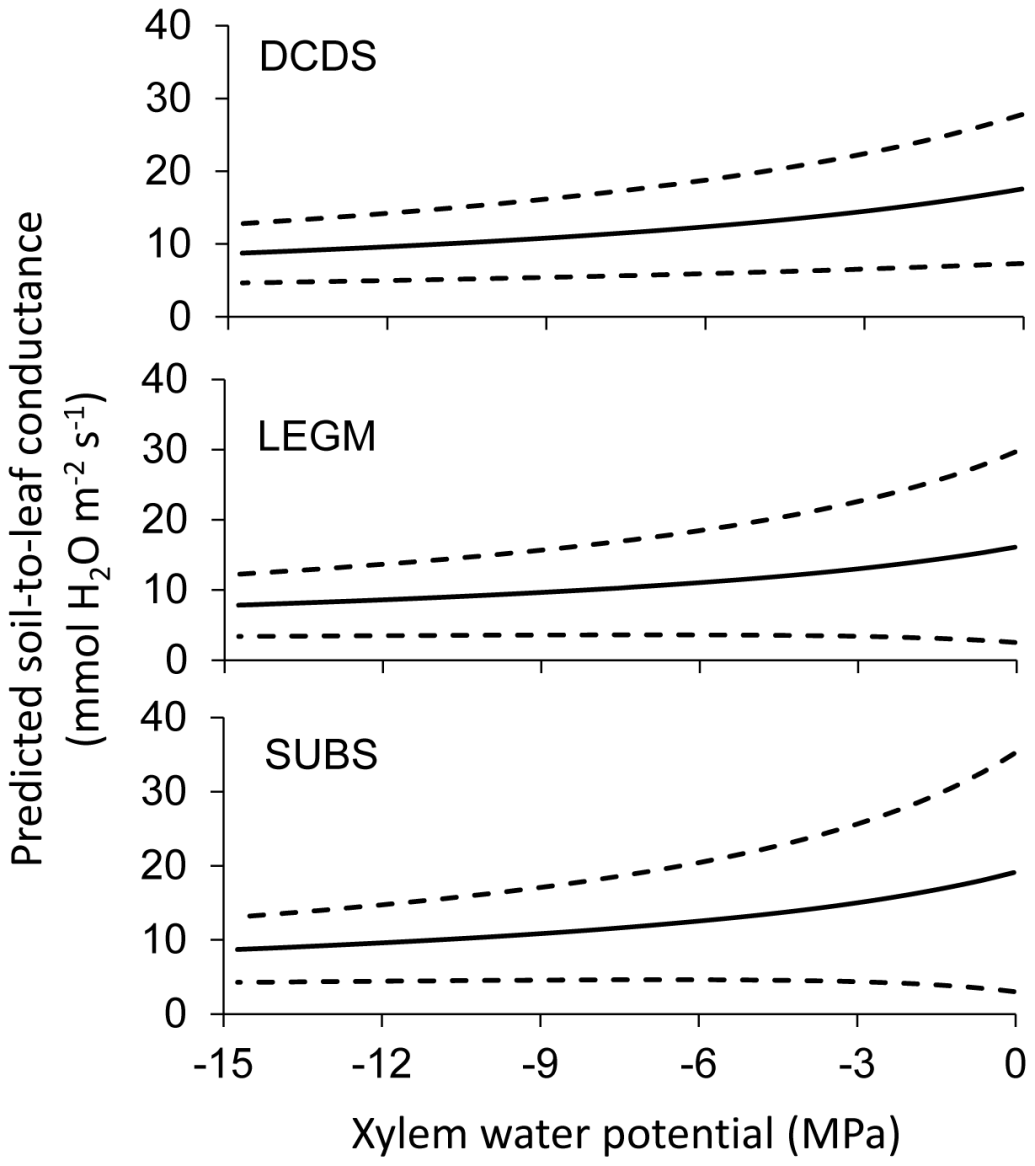
Predicted soil-to-leaf conductance as functions of xylem water potential based on the parameters at the observation level. The solid line stands for the mean, and the broken lines indicate one standard deviation below and above the mean.

Finally the comparison of mean 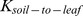 as a function of xylem water potential based on the parameters obtained at the observation level (Fig. 6) shows similar characteristics for the three functional groups. However, the calculated 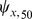 values,at which 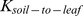 decreased by a half of its maximum value, are −12.3, −14.0, and −16.5 MPa for SUBS, LEGM, and DCDS groups, respectively, indicating that the xylem vessels of the two deciduous shrubs (DCDS and LEGM), on average, are slightly stronger than those of the SUBS.

## Discussion

### Experimental Evidences Connected to the Results

The behavior of stomatal conductance, driven by soil moisture and vapor pressure deficit, depends largely on the stiffness of guard cell structure, and the hydraulic conductance of plant xylem which transports water from soil to leaves. A number of literatures reported the findings of the xylem hydraulic conductivity of various plant life forms.

Kocacinar and Sage [45] experimentally measured xylem hydraulic conductivities of 16 shrub species in west America and central Asia, and found the mean hydraulic conductivities are 3.24×10^−4^ and 0.46×10^−4^ kg m^−1^s^−1^ MPa^−1^ for C_3_ and C_4_ species, respectively. If flow paths are about 2 m long, then the hydraulic conductance would be 9.0 and 1.3 mmol m^−2^ s ^−1^ MPa^−1^ for C_3_ and C_4_ species, respectively. The synthesis of measurements on leaf-specific hydraulic conductivity of various plant growth forms [46] reveals that the leaf-specific whole plant conductance is in the range between 0.2 and 20 mmol m^−2^ s^−1^ MPa^−1^, and that the desert subshrub tends to have higher hydraulic conductance than other growth forms. The measurement of vessel density, diameter, and leaf-specific hydraulic conductance of three *Caragana* species in northern China [47] showed that their soil-to-leaf hydraulic conductance varied between 0.5 to 20 mmol m^−2^ s^−1^ MPa^−1^. The data of Iovi et al. [48] for the Mediterranean species showed that soil-to-leaf conductance varies within a wide range up to 40 mmol m^−2^ s^−1^ MPa^−1^, and the herbaceous group has much higher hydraulic conductance than other groups. They also found a negative exponential decay of the hydraulic conductance with respect to decreased xylem water potential.

Our analysis at the observation level yielded 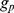 (maximum potential soil-to-leaf conductivity) in the range from 1.04 to 62.13 mmol m^−2^ s^−1^ MPa^−1^, and the parameters of the three shrub functional groups are in the range from 2.05 to 5.66 mmol m^−2^ s^−1^ MPa^−1^, comparable to the above experimental findings. Greater 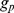 is an indicator of greater vessel lumen diameter, but not an indicator of greater vessel density, as the negative correlation between the two quantities found by Poorter et al. [49]. However, the greater diameter of the vessel members in general means higher potential loss of hydraulic conductivity due to cavitation and embolism of vessel members under xylem tension. This tradeoff is reflected in our obtained 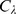 parameter which makes the loss of conductivity proportional to the maximum conductivity. For example, the SUBS group has much higher hydraulic conductance, so that the decrease in conductance with xylem water stress is faster than the other two groups. The result is similar to those found by Iovi et al. [48].

Using finite element analysis, Cooke et al. [50] indicated that a typical stomata aperture width increases from 7 to 15 µm when turgor pressure inside guard cells increases from 0 to 700 kPa. If we assume that stomatal conductance is directly proportional to stomatal aperture and that stomatal apertures of 7 and 15 µm approximately correspond to typical stomatal conductance of 0.3 and 1.0 mol m^−2^ s^−1^, respectively, an approximation of the compliance would be (1.0–0.3)/0.7 = 1.0 mol m^−2^ s^−1^ MPa^−1^. The model parameter 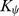 in our analysis is a product of this compliance and stomata density, so that the apparent compliance 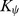 should have more variation range if the variation in stomatal density is considered. We found this parameter varies between 0.088 and 1.482 with a mean of 0.613 at the observation level, and between 0.16 to 1.42 mol m^−2^ s^−1^ MPa^−1^ at the functional group level. The result is comparable to Cook’s finding.

From the findings by Leishman et al. [51], [52], we know that the non-legume deciduous shrubs have lower stomatal conductance than the other two groups. The most deciduous shrubs in this study (both legume and non-legume) were found as xerophyte growing in relatively shallow soils with high water use efficiency. Their instantaneous photosynthesis can be high when soil water is abundant. However, due to the long-term drying conditions, they have to invest large amount of the assimilated products on constructing xylem for small-diameter vessels with lower 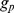, which allows them to sustain the excessive drought conditions. Most legume species are thin-leaved *Caragana* species, which might have something to do with their relatively greater 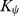 than the non-legume deciduous group.

Finally, the SUBS group (*Artemisia*), with their less lignified stems and leaves, is shown to have the greatest compliance, and the largest potential hydraulic conductance than the two deciduous shrub groups. Thus the subshrubs are shown here to be typical drought-avoiding in most occasions. In this sense, the subshrubs share some characteristics of herbaceous plants, with stomatal conductance more responsive to variation of moistures in soil and air [18], [48]. In other words, the subshrubs behave as an opportunist, so that they have a large stomatal conductance to accommodate the great photosynthesis when moistures are abundant in soil, but choose to close the stomata under severe drought conditions.

### Implication for Macro-scale Ecosystem Dynamics

The dynamics of leaf stomatal conductance is coupled with plant morphological structure and other variables such as leaf nutrient status and boundary layer thickness [28], [30]. The present model should be applied and further tested within more complex ecosystem models to improve its connection with external variables.

Our hierarchical analysis showed that the functional group model is a significant improvement over the global level model, and the model at the observation level is a significant improvement over that at the functional group level. This result suggests the parameters at three levels can be used in ecosystems modeling at large, intermediate, and small scales. At regional and global scales in which we need to place all shrubs in one group, the global level parameters can be used. For small patch-scale ecosystems, parameters at the observation level are more appropriate. Modeling at intermediate mesoscales (watershed or landscape) may necessitate distinguishing the properties among shrubs types, thus the results at the level of functional groups may be applicable.

Our findings at the functional group level offered a clue to the equivocal issue in shrub-grass interaction. The process of shrub encroachment into grasslands has been understood as involving nonlinear processes in soil and plant communities, with strong positive feedbacks that consequently lead to the domination of shrubs in the previous grasslands [53]. The current understanding is that once shrubs get established and dominated in the grasslands, it is impossible for the process to reverse to restore the grasslands because of the altered competition at the community scale. However, recent studies gave a number of exceptional contradictory cases. In a 16-year exclosure (excluding from livestock grazing) established at the Erdos Sandland Ecosystem Station in northern China, it was found that a previously dominating shrub (*Artemisia ordosica* Krasch) decreased by 90% [54], but grasses and forbs increased substantially inside the exclosure. Another example is the *Artemisia frigida* Willd., a commonly observed shrub species in northern China grasslands. The proliferation of this species in the so-called typical steppes has been considered a result of overgrazing [55]. Enclosure studies in northern China showed that this shrub can be put down with appropriate management. Several studies showed that the enclosures with managed livestock grazing kept the dominance of the species less than 8% in the enclosures of 5, 14, and 25 years, with comparison to the 30% dominance in the heavily grazed sites [56], [57], [58]. These results contradict the current understanding of the irreversibility of shrub invasion. However, we noticed that the species in the above case studies are *Artemisia* subshrubs. Our results showed that there is a significant difference in stomatal behavior between the subshrubs and the deciduous shrubs. The deciduous shrubs have great advantages in resistance and tolerance to soil water stress over the grasses, which may contribute to their irreversible encroachment into grasslands. The subshrubs are less resistant and tolerant to soil moisture stress than the shrubs, and thus are less advantageous in the competition with the grasses. Our hypothesis is that when the grazing pressure is heavy, the soil cannot hold enough water because of the decreased grass roots biomass. The subshrubs start to gain dominance. However, when grazing pressure is reduced, root biomass start to increase, allowing fast infiltration of water. Consequently the soil layers tend to hold more water to allow better grass growth, and the advantage of the subshrubs over the grasses is weakened. Therefore, with appropriate management, the invasion of the subshrubs into grasslands might be reversed and the health of the grassland ecosystems might be recovered. This hypothesis has to be tested in more strictly designed experiments.

## Supporting Information

Table S1 Data measured by the authors group with Licor 6400 portable gas analyzer.
(XLSX)
