# Correction: Detecting the Differences in Responses of Stomatal Conductance to Moisture Stresses between Deciduous Shrubs and *Artemisia* Subshrubs

**DOI:** 10.1371/journal.pone.0169382

**Published:** 2016-12-29

**Authors:** Qiong Gao, Mei Yu, Chan Zhou

The affiliation for the first author is incomplete. Qiong Gao is also affiliated with #2 Institute for Tropical Ecosystem Studies, University of Puerto Rico—Rio Piedras, San Juan, Puerto Rico, United States of America.
